# *QuickStats:* Rate* of Alcohol-Induced Deaths^†^ Among Persons Aged ≥25 Years, by Age Group — National Vital Statistics System, 1999–2017

**DOI:** 10.15585/mmwr.mm6833a5

**Published:** 2019-08-23

**Figure:**
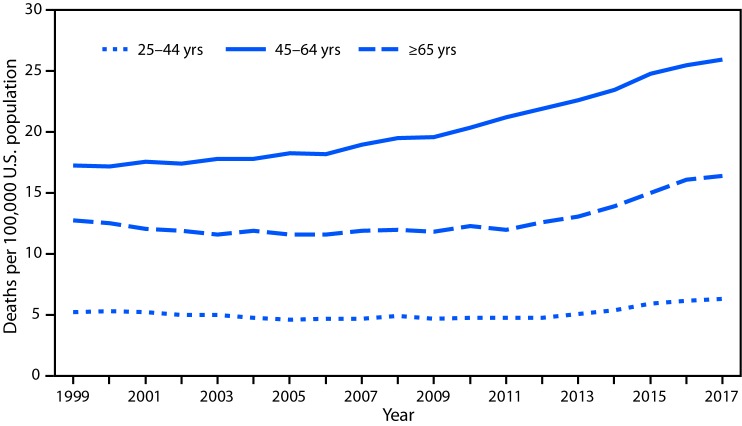
Rates of alcohol-induced deaths for persons aged 45–64 years increased from 17.3 per 100,000 population in 1999 to 26.0 in 2017. For persons aged 25–44 years, rates declined from 1999 to 2005, were stable from 2005 to 2012, and then increased from 2012 (4.8) to 2017 (6.3). A similar pattern was observed for persons aged ≥65 years, with an initial decline, a stable period, and then an increase from 2011 (12.0) to 2017 (16.4).

**Source:** National Vital Statistics System, Mortality Data, 1999–2017. https://www.cdc.gov/nchs/nvss/deaths.htm.

